# Phase-separated protein droplets of amyotrophic lateral sclerosis-associated p62/SQSTM1 mutants show reduced inner fluidity

**DOI:** 10.1016/j.jbc.2021.101405

**Published:** 2021-11-12

**Authors:** Mohammad Omar Faruk, Yoshinobu Ichimura, Shun Kageyama, Satoko Komatsu-Hirota, Afnan H. El-Gowily, Yu-shin Sou, Masato Koike, Nobuo N. Noda, Masaaki Komatsu

**Affiliations:** 1 Department of Physiology, Juntendo University Graduate School of Medicine, Bunkyo-ku, Tokyo, Japan; 2 Department of Cell Physiology, Niigata University Graduate School of Medical and Dental Sciences, Chuo-ku, Niigata, Japan; 3 Biochemistry Division, Chemistry Department, Faculty of Science, Tanta University, Tanta, Egypt; 4 Department of Cell Biology and Neuroscience, Juntendo University Graduate School of Medicine, Bunkyo-ku, Tokyo, Japan; 5 Laboratory of Structural Biology, Institute of Microbial Chemistry (BIKAKEN), Shinagawa-ku, Tokyo, Japan

**Keywords:** p62, liquid droplet, amyotrophic lateral sclerosis, autophagy, NRF2, ALS, amyotrophic lateral sclerosis, BRET, bioluminescence resonance energy transfer, FTD, frontotemporal degeneration, GSTM1, Glutathione S-transferase Mu 1, KIR, Kelch-like ECH-associated protein 1–interacting region, LIR, LC3-interacting region, mTORC1, mechanistic target of rapamycin complex 1, NQO1, NAD(P)H dehydrogenase, quinone 1, NRF2, nuclear factor erythroid 2–related factor 2, PDB, Paget’s disease of bone, RBP, RNA-binding protein, TRAF6, tumor necrosis factor receptor–associated factor 6, UBA, ubiquitin-associated, UGDH, UDP-glucose 6-dehydrogenase

## Abstract

Several amyotrophic lateral sclerosis (ALS)-related proteins such as FUS, TDP-43, and hnRNPA1 demonstrate liquid–liquid phase separation, and their disease-related mutations correlate with a transition of their liquid droplet form into aggregates. Missense mutations in *SQSTM1/p62*, which have been identified throughout the gene, are associated with ALS, frontotemporal degeneration (FTD), and Paget’s disease of bone. SQSTM1/p62 protein forms liquid droplets through interaction with ubiquitinated proteins, and these droplets serve as a platform for autophagosome formation and the antioxidative stress response *via* the LC3-interacting region (LIR) and KEAP1-interacting region (KIR) of p62, respectively. However, it remains unclear whether ALS/FTD-related p62 mutations in the LIR and KIR disrupt liquid droplet formation leading to defects in autophagy, the stress response, or both. To evaluate the effects of ALS/FTD-related p62 mutations in the LIR and KIR on a major oxidative stress system, the Keap1-Nrf2 pathway, as well as on autophagic turnover, we developed systems to monitor each of these with high sensitivity. These methods such as intracellular protein–protein interaction assay, doxycycline-inducible gene expression system, and gene expression into primary cultured cells with recombinant adenovirus revealed that some mutants, but not all, caused reduced NRF2 activation and delayed autophagic cargo turnover. In contrast, while all p62 mutants demonstrated sufficient ability to form liquid droplets, all of these droplets also exhibited reduced inner fluidity. These results indicate that like other ALS-related mutant proteins, p62 missense mutations result in a primary defect in ALS/FTD *via* a qualitative change in p62 liquid droplet fluidity.

*SQSTM1*, the human gene encoding p62/SQSTM1 (hereafter referred to as p62), is localized on chromosome 5 and comprises eight exons up to 16 kb in length. Missense mutations in *SQSTM1* have been primarily associated with amyotrophic lateral sclerosis (ALS), frontotemporal lobar degeneration (FTD), and Paget’s disease of bone (PDB) (1, 2). Various p62-positive structures, including inclusion bodies, have been identified in patients with neurodegenerative diseases such as ALS and FTD (3, 4, 5). However, the common mechanism whereby different mutations in the gene encoding p62 cause ALS and FTD remains unclear.

p62, initially identified as a 62-kDa protein, has multiple functional domains that include the N-terminal Phox1 and Bem1p (PB1) domains, a zinc finger (ZZ), a tumor necrosis factor receptor–associated factor 6 (TRAF6) binding (TB) motif, an LC3-interacting region (LIR), a Kelch-like ECH-associated protein 1 (KEAP1)-interacting region (KIR), and a ubiquitin-associated (UBA) domain (6, 7). The protein is not localized only in the cytoplasm, but can also be observed in the nucleus, in autophagosomes, and in lysosomes (8, 9, 10). Due to its role as an adaptor for selective autophagy, p62 also localizes to cargos that will be degraded, such as ubiquitin-positive protein aggregates, damaged mitochondria, and invading bacteria (11). Under certain circumstances, p62 can also be degraded by endosomal microautophagy (12), but like ubiquitinated cargos, it is primarily degraded during selective autophagy through interaction with FIP200/RB1-inducible coiled-coil protein 1, an upstream factor for autophagosome formation, and the subsequent mutually exclusive interaction with LC3, an autophagosome-localizing protein (13, 14, 15). p62 is also known as a multifunctional signaling hub, as it participates not only in its aforementioned adaptor role in selective autophagy, but also in the activation of mechanistic target of rapamycin complex 1 (mTORC1) during nutrient sensing and NF-κB activation during inflammation, apoptosis, and activation of the Keap1-Nrf2 pathway for antioxidant response (16). Among these, the p62-mediated nuclear factor erythroid 2–related factor 2 (NRF2) pathway is specifically coupled with selective autophagy. NRF2 is a basic leucine zipper transcription factor, and its heterodimer with small MAF proteins controls the expression of proteins that protect against oxidative damage triggered by injury and inflammation. The tumor suppressor KEAP1 is an adaptor of cullin3-based ubiquitin ligase for NRF2. When p62 interacts with ubiquitinated substrates, they condense to form p62 bodies (17, 18). Thereafter, Ser349 of p62 in the condensates is phosphorylated by multiple kinases including mTORC1 and transforming growth factor-beta-activated kinase 1 (19), which enhances the binding to KEAP1 (20, 21). Thus, KEAP1 is sequestered in the condensates, and NRF2 escapes from the KEAP1 interaction and translocates into the nucleus to induce the gene expression of a battery of NRF2 targets. To degrade the ubiquitinated substrates and suppress the activation of NRF2, p62 bodies are degraded by autophagy (8, 22, 23).

Growing lines of evidence have recently shed light on a unique feature of p62, namely phase separation of p62 that is dependent on the presence of ubiquitin chains (17). Phase-separated p62 droplets allow for exchange of their components, including ubiquitin, LC3, and KEAP1, with the surrounding environment (17, 18, 21, 24). Inside liquid-like droplets, molecules are predicted to maintain their conformation and activity. Consequently, such droplets could also serve as nodes from which signaling cascades could be activated in the context of selective autophagy and the Keap1-Nrf2 pathway (21, 25). p62 clustering appears to require multiple ubiquitin chains that each contain at least three ubiquitin moieties. Various ubiquitin chain linkages, but especially K63, are able to promote clustering, whereas free monoubiquitin or unanchored ubiquitin chains, specifically the K48 linkage, inhibit p62 clustering (18). These p62 structures arise from existing p62 filaments that are cross-linked by polyubiquitinated substrates (25, 26). Inside these clustered droplets, p62 appears to retain little mobility, whereas ubiquitin, LC3, and KEAP1 may be able to diffuse more easily within the cluster and the surrounding cytosol (17, 18, 21). Finally, cluster formation is rendered more efficient by the presence of NBR1, another autophagic adaptor that cooperates with p62 in cells (18, 24). Thus, p62 droplets and/or gels are not simple substrates for autophagy but serve as platforms for both autophagosome formation and the antioxidative stress response (21, 25).

Some ALS/FTD-related LIR or KIR p62 mutants, but not all, showed decreased NRF2 activation or delayed autophagic degradation (Table 1). In this study, we aimed to identify the structural or biochemical basis for functional deficits associated with disease-relevant p62 mutations.

**Table 1:** Functional defects of p62 mutants as reported in previous studies

p62 mutation (related diseases)	KEAP1 interaction (NRF2 activation)	LC3 interaction (autophagic degradation of p62)	Ref.
D336N (ALS/FTD, PDB)	n.d. (n.d.)	n.d. (n.d.)	−
D337E (ALS/FTD)	n.d. (n.d.)	+ (n.d.)	(27, 28)
L341V (ALS)	+ (+)	− (−)	(28, 29)
K344E (ALS/FTD)	+ (+)	n.d. (n.d.)	(29)
P348L (ALS/FTD)	− (−)	n.d. (n.d.)	(29)
S349T (PDB)	− (−)	n.d. (n.d.)	(30)
G351A (ALS/FTD)	− (−)	n.d. (n.d.)	(29)

n.d.: not determined., −: weaker interaction or less degradation than wild-type p62, +: interaction or degradation comparable to wild-type p62. c.1006G > A (p.Asp336Asn) was found in ClinVar (https://www.ncbi.nlm.nih.gov/clinvar/variation/542160/).

## Results

### Models for binding of disease-related p62 mutants with LC3 and KEAP1

**Figure 1:**
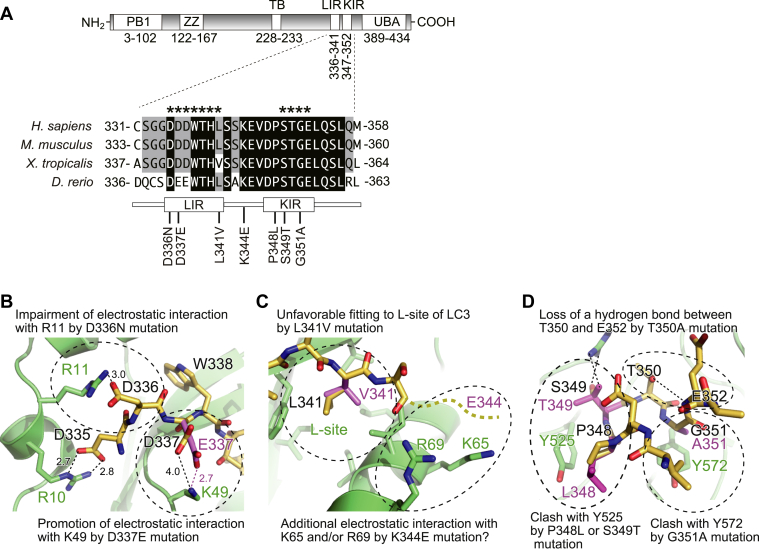
**Models for binding of disease-related p62 mutants with LC3 and KEAP1.***A*, domain structure of p62 and disease-related mutations in the LIR and KIR. *B*, effects of D336N and D337E mutations on interaction with LC3. The side chain of Glu337 was manually modeled. *Numbers* indicate distance in Å. *C*, effects of L341V and K344E mutations on interaction with LC3. The side chain of Val341 was manually modeled. Figures in (*B*) and (*C*) were prepared using the crystal structure of the LC3B-p62 LIR complex (PDB 2ZJD). Residue numbers refer to human proteins. *D*, effects of P348L, T350A, S349T, and G351A mutations on interaction with KEAP1. Figure in (*D*) was prepared using the crystal structure of the KEAP1-p62 KIR complex (PDB 3ADE). Residue numbers refer to human proteins. KIR, KEAP1-interacting region; LIR, LC3-interacting region; PB1, Phox and Bem1p; TB, TRAF6-binding domain; UBA, Ubiquitin-associated domain; ZZ, Zinc finger.

Disease-related p62 mutants, which have missense mutations located between the LIR and KIR (Fig. 1*A*), have been investigated in terms of their effects on selective autophagy and p62-mediated NRF2 activation (Table 1), but no consistent effect of these mutations has been identified. We previously clarified the binding modes of the LIR with LC3 (PDB 2ZJD) (14) and of the KIR with KEAP1 (PDB 3ADE) (22). On the basis of these structural data, we constructed models of complexes formed between p62 mutants and either LC3 or KEAP1 (Fig. 1, *B*–*D*). As shown in Figure 1*B*, p62 Asp336 forms an electrostatic interaction with LC3 Arg11. Loss of a negative charge due to D336N mutation weakens this interaction and thereby reduces the affinity of p62 for LC3. On the other hand, D337E mutation strengthens the interaction with LC3 by creating an electrostatic interaction between Glu337 and Lys49 (Fig. 1*B*) (Asp337 is located distally from Lys49 and thus cannot form an electrostatic interaction). The side chain of Leu341 is bound to the L-site of LC3, and the binding affinity becomes lower by L341V mutation due to poor fit (Fig. 1*C*). P348L, S349T, and G351A mutations weaken the interaction with KEAP1 by causing a steric clash with Tyr525 (P348L and S349T) or Tyr572 (G351A) of KEAP1 (Fig. 1*D*). On the other hand, T350A mutation may indirectly weaken the interaction with KEAP1 by the following mechanism: the side chain of Thr350 forms an intramolecular hydrogen bond with the main chain of Glu352 (Fig. 1*D*); T350A mutation impairs this hydrogen bond, which may cause a conformational change in p62 KIR and thus lead to reduced affinity for KEAP1.

### *In vitro* interaction of disease-related p62 mutants with FIP200, LC3, and KEAP1

**Figure 2:**
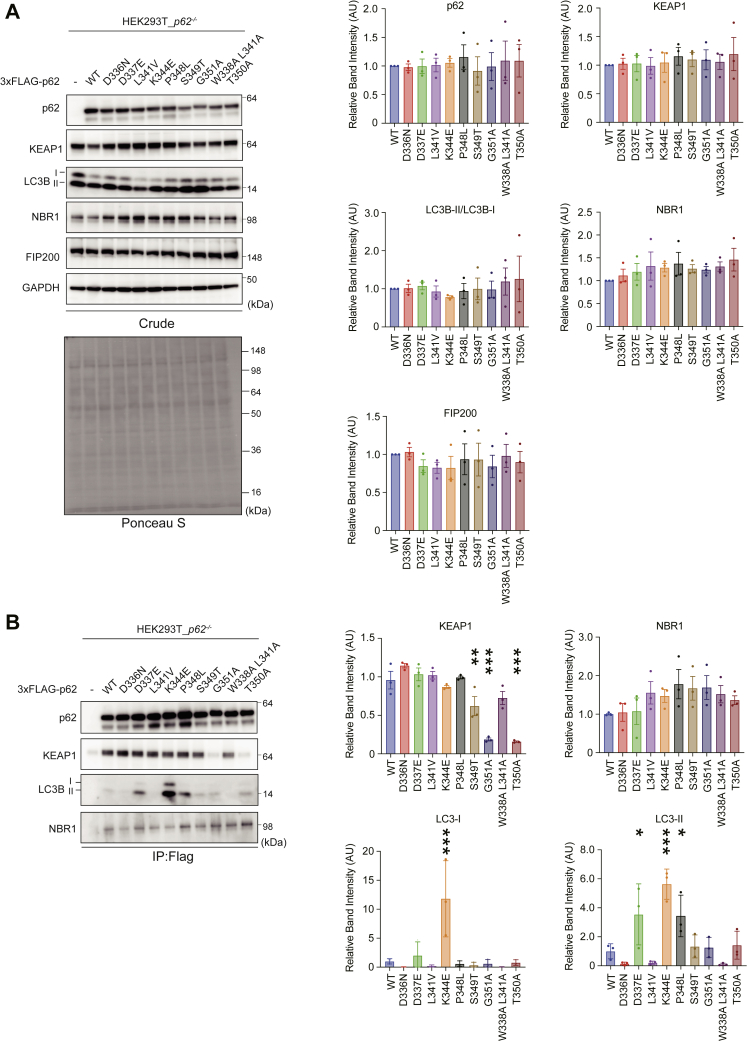
**Interaction of disease-related p62 mutants with their binding partners.***A*, immunoblot analysis. FLAG-tagged wild-type and mutant p62 were expressed in *p62*^*−/−*^ HEK293T cells. At 24 h after transfection, cell lysates were prepared and subjected to immunoblot analysis with the indicated antibodies. Data shown are representative of four separate experiments. Bar graphs show the results of quantitative densitometric analysis of the indicated proteins relative to whole proteins, as estimated by Ponceau-S staining (n = 4). Data are means ± s.d. Statistical analysis was performed by Dunnett’s test after ANOVA. Significant differences are shown for values of mutant p62-expressing cells and wild-type p62-expressing cells. *B*, immunoprecipitation assay. Cell lysates prepared as described in (*A*) were immunoprecipitated with FLAG antibody, and the immunoprecipitants were subjected to immunoblot analysis with the indicated antibodies. Data shown are representative of three separate experiments. Bar graphs show the results of quantitative densitometric analysis of the indicated proteins (n = 3). Data are means ± s.d. ∗*p* < 0.05, ∗∗*p* < 0.01 and ∗∗∗*p* < 0.001 as determined by Dunnett’s test after ANOVA. Significant differences are shown for values of mutant p62-expressing cells and wild-type p62-expressing cells.

To test whether disease-related p62 mutant proteins affect selective autophagy and the Keap1-Nrf2 system, we generated *p62*-deficient HEK293T cells (Fig. S1) and expressed each FLAG-tagged wild-type and p62 mutant. Subsequently, the cell lysates were subjected to SDS-PAGE, followed by immunoblot analysis. Expression of p62 mutant proteins did not influence KEAP1 or FIP200 levels or the conversion of LC3B-I to LC3B-II, the latter of which is an indicator for autophagy induction (Fig. 2*A*). An immunoprecipitation assay with anti-FLAG antibody showed that all mutants, except for disease-related mutants p62^S349T^and p62^G351A^, and KEAP1-binding defective p62^T350A^ (22), demonstrated binding affinity to KEAP1 that was comparable to wild-type p62 (Fig. 2*B*). The immunoprecipitate prepared in our experimental settings did not contain FIP200 (data not shown). Consistent with the structural models (Fig. 1, *B* and *C*), p62^D337E^ and p62^K344E^ mutants showed higher affinity to the LC3-II form (Fig. 2*B*). The affinity of p62^P348L^ to LC3-II was also higher (Fig. 2*B*). Our immunoprecipitation assay was not completely consistent with previous reports (27, 28, 29, 30) (Tables 1 and 2). While immunoprecipitation assays performed by Layfield’s group showed that P348L, S349T, and G351A had less ability to bind to endogenous KEAP1 (Table 1), we observed that P348L still bound to endogenous KEAP1. The experimental settings in the two studies were almost identical except for genotype. While they used wild-type HEK293T cells, we used *p62*-deficient HEK293T cells. p62 forms homo-oligomers through the N-terminal PB1 domain. Thus, when using wild-type HEK293T cells, endogenous p62 may affect the interaction of P348L with LC3 and/or KEAP1.

**Table 2:** Summary of interactions of p62 mutants with KEAP and LC3

p62 mutation (related diseases)	KEAP1 interaction (IP and NanoBRET)	LC3 interaction (IP and NanoBRET)	Autophagic degradation	Colocalization (KEAP1 and LC3B)
D336N (ALS/FTD, PDB)	+ and +	+ and +	−	+ and –
D337E (ALS/FTD)	+ and +	+ + and +	+	+ and +
L341V (ALS)	+ and +	+ and −	+	+ and +
K344E (ALS/FTD)	+ and +	+ + and +	+	+ and +
P348L (ALS/FTD)	+ and −	+ + and +	+	− and +
S349T (PDB)	− and +	+ and +	+	+ and +
G351A (ALS/FTD)	− and −	+ and +	+	− and +
W338A L341A (−)	+ and +	+ and −	−	+ + and –
T350A (−)	− and −	+ and +	+	− and +

−: weaker interaction, less autophagic degradation, or a lower colocalization rate than wild-type p62, +: comparable to wild-type p62, ++: higher affinity or a higher colocalization rate than wild-type p62.

### *In vivo* interaction of disease-related p62 mutants with FIP200, LC3, and KEAP1

**Figure 3:**
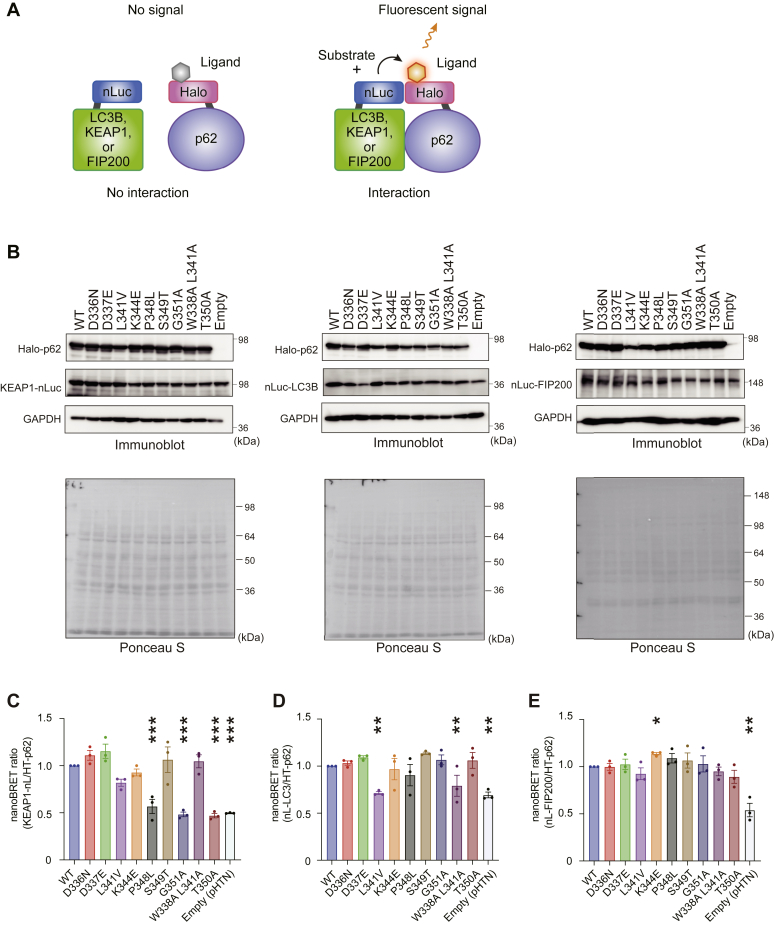
***In vivo* interaction of disease-related p62 mutants with their binding partners.***A*, principle of NanoBRET assay. *B*, immunoblot analysis. Each HaloTag-tagged wild-type and mutant p62 was coexpressed with NanoLuc Luciferase-tagged KEAP1, LC3, or FIP200 in *p62*^*−/−*^ HEK293T cells. At 24 h after transfection, cell lysates were prepared and subjected to immunoblot analysis with anti-p62, anti-KEAP1, and anti-GAPDH antibodies. Data shown are representative of four separate experiments. *C*–*E*, NanoBRET assays of p62 and KEAP1 (*C*), LC3B (*D*), and FIP200 (*E*). Bar graphs show the results of quantitative analysis of the indicated mutant p62 proteins relative to wild-type p62 (n = 3). Data are means ± s.d. ∗*p* < 0.05, ∗∗*p* < 0.01, and ∗∗∗*p* < 0.001 as determined by Dunnett’s test after ANOVA. Significant differences are shown for values of mutant p62-expressing cells and wild-type p62-expressing cells.

Cellular p62 takes the form of a droplet/gel (21). Immunoprecipitation assays are suitable for evaluating protein–protein interactions in noncellular systems. Since these assays are usually performed in the presence of one or more detergents, such as Triton X-100, they not suitable for detecting the association between droplets and intracellular proteins. To investigate the effect of p62 mutations on the affinity of p62 to its binding partners in cells, we conducted a bioluminescence resonance energy transfer (BRET)-based assay that uses NanoLuc Luciferase as the BRET energy donor and HaloTag protein labeled with the HaloTag NanoBRET 618 fluorescent ligand as the energy acceptor to measure the interaction of two binding partners in live cells (31) (Fig. 3*A*). NanoLuc Luciferase was fused with LC3B, one of three LC3 homologues, and FIP200 at the N-terminus, and with KEAP1 at the C-terminus. p62 was fused with HaloTag at the N-terminus. These constructs were transfected into *p62*-deficient HEK293T cells, and we confirmed efficient expression of each protein by immunoblot analysis (Fig. 3*B*). The NanoBRET assay revealed interaction features of p62 that were different from those demonstrated in the immunoprecipitation assay (Fig. 3, *C*–*E*). While the immunoprecipitation assay showed that all disease-related mutants except for p62^S349T^ and p62^G351A^ interacted with KEAP1 to the same extent (Fig. 2*B*), the NanoBRET assay revealed that p62^P348L^ also had lower binding affinity for KEAP1 (Fig. 3*C*), which is consistent with structural models (Fig. 1*D*). By contrast, while p62^S349T^ showed defective binding to KEAP1 in the immunoprecipitation assay, in the NanoBRET assay it exhibited similar binding affinity to KEAP1 as wild-type p62 (Fig. 3*C*). Regarding the interaction of p62 with LC3B, p62^L341V^ and p62^W338A L341A^ showed lower binding affinity to LC3B than wild-type p62 (Fig. 3*D*). Unlike in the immunoprecipitation assay, no p62 mutants showed increased binding affinity to LC3B in the NanoBRET assay (Fig. 3*D*). Though we did not detect any interaction of p62 with FIP200 in the immunoprecipitation assay (data not shown), this interaction was detectable in the NanoBRET assay (Fig. 3*E*). The p62^K344E^ mutant exhibited a slightly but statistically significant increase in the interaction with FIP200 (Fig. 3*E*). Unlike the immunoprecipitation assay, the NanoBRET assay can detect the binding of p62 gels to intracellular proteins. Therefore, the P348L mutant showed weaker binding to KEAP1 only in the NanoBRET assay and may have defective interaction with KEAP1 when the mutant is condensed. Likewise, p62 gels consisting of L341V but not monomer L341V may have decreased binding affinity to LC3. Since p62 gels contain NBR1 and Tax1BP1 that interact with LC3 and concomitantly affect the association of KEAP1 with these gels (24, 32), it is plausible that they positively or negatively affect the *in vivo* interaction of p62 with KEAP1 and LC3.

### The effects of p62 mutants on p62-mediated NRF2 activation

**Figure 4:**
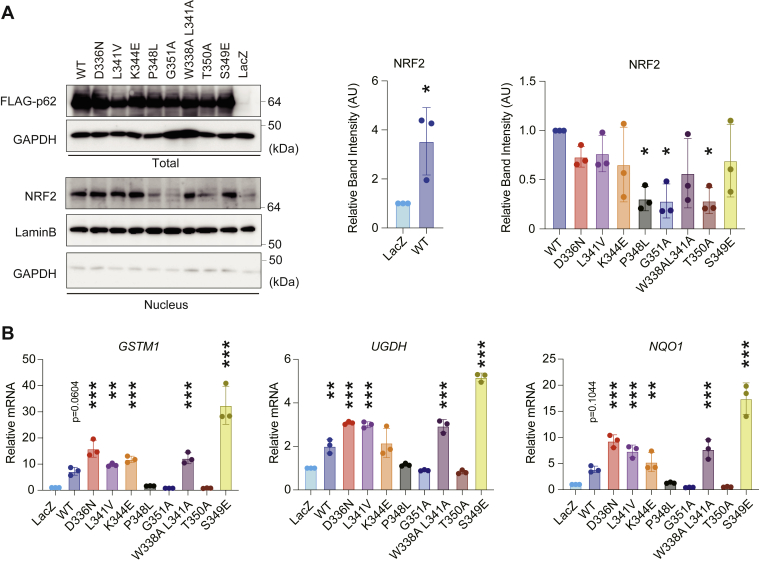
**NRF2 activation by disease-related p62.***A*, immunoblot analysis. Wild-type p62 and each p62 mutant were expressed in primary mouse hepatocytes using an adenovirus system. At 48 h after infection, the cells were fractionated into cytosolic and nuclear fractions, which were subjected to immunoblot analysis with anti-FLAG, anti-GAPDH, anti-NRF2, and anti-lamin B antibodies. Bar graphs show the results of quantitative densitometric analysis of nuclear NRF2 (n = 3) relative to lamin B (n = 3). To confirm the purity of the nuclear fraction, we conducted an immunoblot analysis with the nuclear fraction using anti-GAPDH antibody. *Left graph*: data are means ± s.d. ∗*p* < 0.05 as determined by two-sided Welch’s *t* test. *Right graph*: data are means ± s.d. ∗*p* < 0.05 as determined by Dunnett’s test after ANOVA. Significant differences are shown for values of wild-type p62-expressing cells compared with those of LacZ expressing cells (*left graph*) and for values of mutant p62-expressing cells compared with those of wild-type expressing cells (*right graph*). *B*, gene expression of NRF2 targets. Total RNAs were prepared from primary mouse hepatocytes expressing LacZ (n = 3), wild-type p62 (n = 3), and each p62 mutant (n = 3). Values were normalized against the amount of mRNA in LacZ-expressing hepatocytes. RT qPCR analyses were performed as technical replicates on each biological sample. Data are means ± s.d. ∗∗*p* < 0.01 and ∗∗∗*p* < 0.001 as determined by Dunnett’s test after ANOVA. Significant differences are shown for values of wild-type and mutant p62-expressing cells compared with those of LacZ expressing cells.

p62 acts as a positive regulator of NRF2 through direct interaction with KEAP1 (20, 22). The stress response mediated by NRF2 occurs in all tissues, but it plays pivotal roles in the liver, where it is responsible for the production of phase-I phase-II drug-metabolizing enzymes and efflux transporters (33). To evaluate the effect of each mutation on p62-mediated NRF2 activation with high sensitivity, we developed adenovirus vectors, which are able to express target proteins effectively even in primary cultured cells, for wild-type human p62 and each mutant. We infected them with primary mouse culture hepatocytes and confirmed their successful expression (Fig. 4*A*). We used the p62^S349E^ mutant, a phosphomimic mutant for S349-phosphorylated p62 that is involved in NRF2 activation, as the positive control, and the KEAP1-binding defective p62^T350A^ as the negative control (20, 21). In comparison with lacZ-expressing primary hepatocytes, the amount of nuclear NRF2 protein increased following expression of wild-type p62 (Fig. 4*A*). The levels of nuclear NRF2 protein in cells expressing p62^P348L^, p62^G351A^, and p62^T350A^ were much lower than those in wild-type p62-expressing cells (Fig. 4*A*). In agreement with these results, real-time PCR analysis revealed that the gene expression of NRF2 targets such as *Glutathione S-transferase Mu 1* (*GSTM1*), *UDP-glucose 6-dehydrogenase* (*UGDH*), and *NAD(P)H dehydrogenase, quinone 1* (*NQO1*), was induced by the expression of wild-type p62, particularly in the case of *UGDH*. This induction did not occur in hepatocytes expressing P348L, G351A, or T350A mutants (Fig. 4*B*). No abnormalities in nuclear NRF2 levels or in the induction of NRF2 target genes were associated with the expression of other disease-related mutants (Fig. 4, *A* and *B*).

### The effects of p62 mutants on their own autophagic turnover

**Figure 5:**
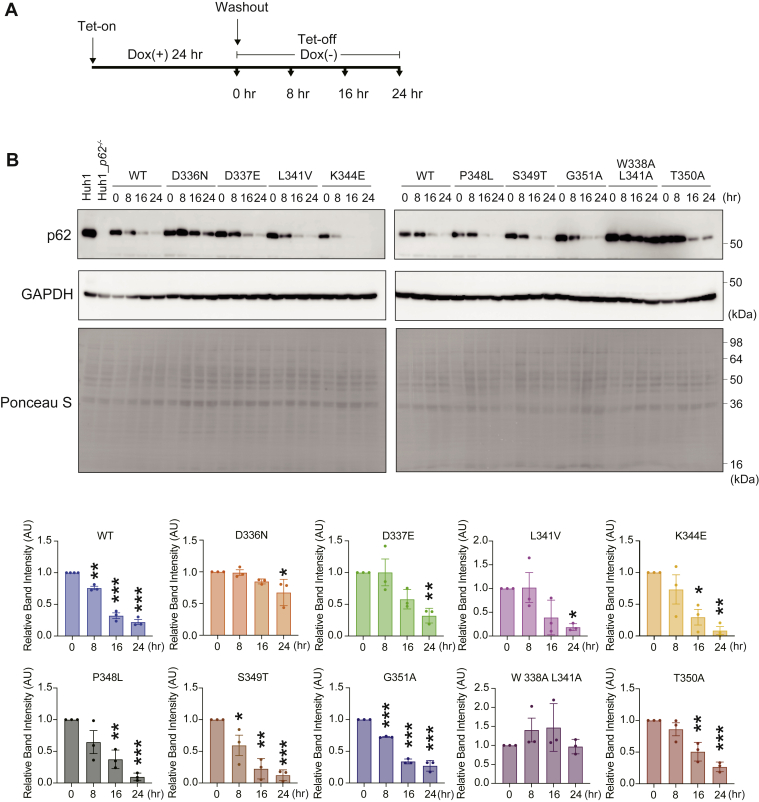
**Autophagic degradation of disease-related p62.***A*, schematic representation of experiments conducted to monitor p62 protein levels. *B*, immunoblot analysis. FLAG-p62 and each disease-related mutant were expressed in *p62*-knockout Huh-1 cells as a result of treatment with doxycycline (Dox) for 24 h and then cultured with media without Dox for the indicated times. Cell lysates were prepared and subjected to SDS-PAGE followed by immunoblotting with the indicated antibodies. Data are representative of three separate experiments. Bar graphs show the results of quantitative densitometric analysis of the indicated proteins relative to whole proteins, as estimated by Ponceau-S staining (n = 3). Data are means ± s.d. ∗*p* < 0.05, ∗∗*p* < 0.01, and ∗∗∗*p* < 0.001 as determined by Dunnett’s test after ANOVA. The p62 protein level at 0 h after removal of Dox was compared with the values at 8, 16, and 24 h.

In the next series of experiments, we investigated the lysosomal degradation of each disease-related p62 mutant protein, since it is possible that mutations around the LIR affect the interaction of each protein with LC3 and subsequently these proteins’ autophagic degradation. To do this, we developed p62-knockout Huh-1 cells (Fig. S1) and transduced doxycycline-inducible wild-type or each disease-related mutant of *p62* gene with retroviral vector. To monitor the half-life of wild-type p62 and each p62 mutant, FLAG-tagged proteins were expressed by the treatment of doxycycline for 24 h, and then the cells were cultured in medium without doxycycline for the indicated time (Fig. 5*A*). While the amount of wild-type FLAG-p62 decreased by approximately 70% at 24 h after removal of doxycycline, that of p62^W338A L341A^ remained unchanged (Fig. 5*B*). The level of p62^D336N^ remained high, though it slightly but significantly decreased at 24 h after the removal of doxycycline (Fig. 5*B*). Unexpectedly, the L341V mutant, which exhibited the loss of binding ability to LC3 in the NanoBRET assay (Fig. 3*D*), was degraded to the same extent as wild-type p62 (Fig. 5*B*). Likewise, the levels of other disease-related mutants, including p62^D337E^, p62^K344E^, p62^P348L^, p62^S349T^, and p62^G351A^, decreased significantly after removal of doxycycline (Fig. 5*B*). An autophagy flux assay with bafilomycin A_1_ (Baf A_1_), which is an inhibitor of lysosomal acidification, revealed that while the levels of wild-type p62 and most mutant p62 proteins increased following treatment with Baf A_1_, this effect was not observed with the p62^D336N^ and p62^W338A L341A^ mutants (Fig. S2), indicating that these mutants were resistant to autophagy. Meanwhile, neither wild-type p62 nor any p62 mutant had any effect on the lysosomal degradation of NBR1 or LC3-II (Fig. S2), both of which are substrates for autophagy.

### Disease-related p62 mutants form liquid droplets

**Figure 6:**
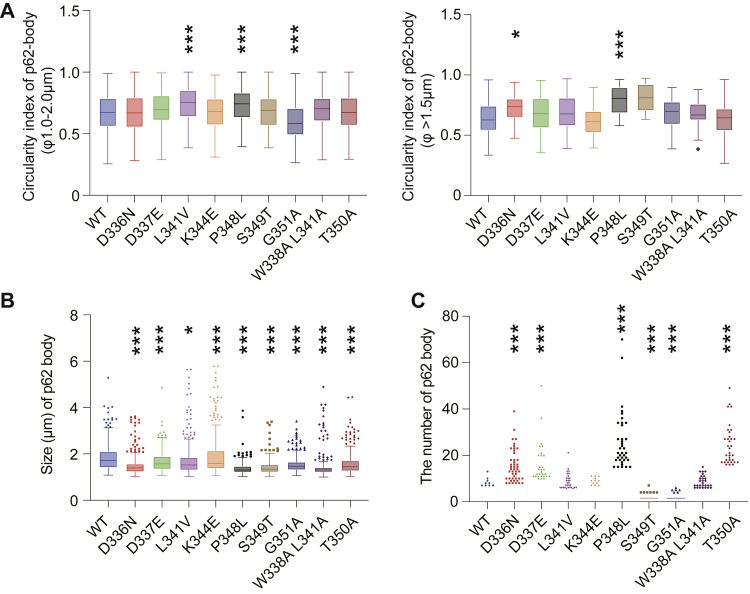
**Formation of droplets consisting of disease-related p62.***A*–*C*, circularity (*A*), size (*B*), and number (*C*) of p62 structures. FLAG-p62 or each disease-related mutant protein was expressed in *p62*-knockout Huh-1 cells using an adenovirus system. The circularity, size, and number of FLAG-p62 structures were determined as described in Experimental procedures. *Graphs* indicate the quantitative analysis of circularity (n = 200), size (n = 300), and number (n = 300). Data are means ± s.d. ∗*p* < 0.05 and ∗∗∗*p* < 0.001 as determined by Dunnett’s test after ANOVA. Significant differences are shown between values for droplets in mutant p62-expressing cells and those in wild-type expressing cells.

Mutants of p62 that cause the same disease(s) were associated with separate or no defects in NRF2 activation or their own autophagic turnover (Figs. 4 and 5). To clarify end points where the p62 mutations converge, we investigated whether mutations of p62 in the LIR and KIR affected the cellular localization of p62 and/or the dynamics of p62 structure. Each GFP-tagged wild-type and mutant p62 was expressed in *p62*-deficient Huh-1 cells by treatment with doxycycline for 48 h, and GFP-p62 signals were observed. A number of GFP-p62-positive structures were present regardless of the presence or absence of mutations. We first measured the circularity of the p62 structures. The closer the circularity is to 1, the greater the ability of the structure to form a liquid droplet. As shown in Figure 6*A*, the circularity of several mutant p62 structures was closer to 1 than that of wild-type p62 structures, implying higher circularity. We also did not observe lower circularity of any mutant p62 structures with a diameter over 1.5 μm (Fig. 6*A*), suggesting that the disease-related mutants become liquid droplets. Time-lapse imaging showed that p62 structures with or without mutations moved through the cytoplasm and occasionally fused with each other (Movies S1–S10), thus meeting a criterion for liquid droplets. We also evaluated the size and number of p62 liquid droplets (Fig. 6, *B* and *C*), as summarized in Table 3. Droplets consisting of any of the p62 mutants were smaller than those of wild-type p62 droplets (Fig. 6*B*). There were fewer droplets in cells expressing p62^S349T^ or p62^G351A^ than in wild-type p62-expressing cells (Fig. 6*C*), suggesting that these two mutants were less able to form liquid droplets.

**Table 3:** Summary of p62 liquid droplet circularity, number, size, inner fluidity, and colocalization with LC3 and KEAP1

p62 mutation (related diseases)	Circularity (1–2 μm and >1.5 μm)	Size and number	Inner fluidity (*t*_*50*_ and mobile fraction)	Colocalization (KEAP1 and LC3B)
D336N (ALS/FTD, PDB)	+ and + +	− and + +	+ and –	+ and –
D337E (ALS/FTD)	+ and +	− and + +	+ and –	+ and +
L341V (ALS)	+ + and +	− and +	+ and –	+ and +
K344E (ALS/FTD)	+ and +	− and +	+ and –	+ and +
P348L (ALS/FTD)	+ + and + +	− and + +	− and −	− and +
S349T (PDB)	+ and +	− and −	+ and –	+ and +
G351A (ALS/FTD)	+ + and +	− and −	+ and –	− and +
W338A L341A (−)	+ and +	− and +	+ and –	+ + and –
T350A (−)	+ and +	− and + +	+ and –	− and +

−: smaller size, larger number, slower T50, smaller mobile fraction, or lower colocalization rate than wild-type p62, +: comparable to wild-type p62, ++: higher circularity, larger number, or higher colocalization rate than wild-type p62.

### Reduced inner fluidity of disease-related p62 liquid droplets

**Figure 7:**
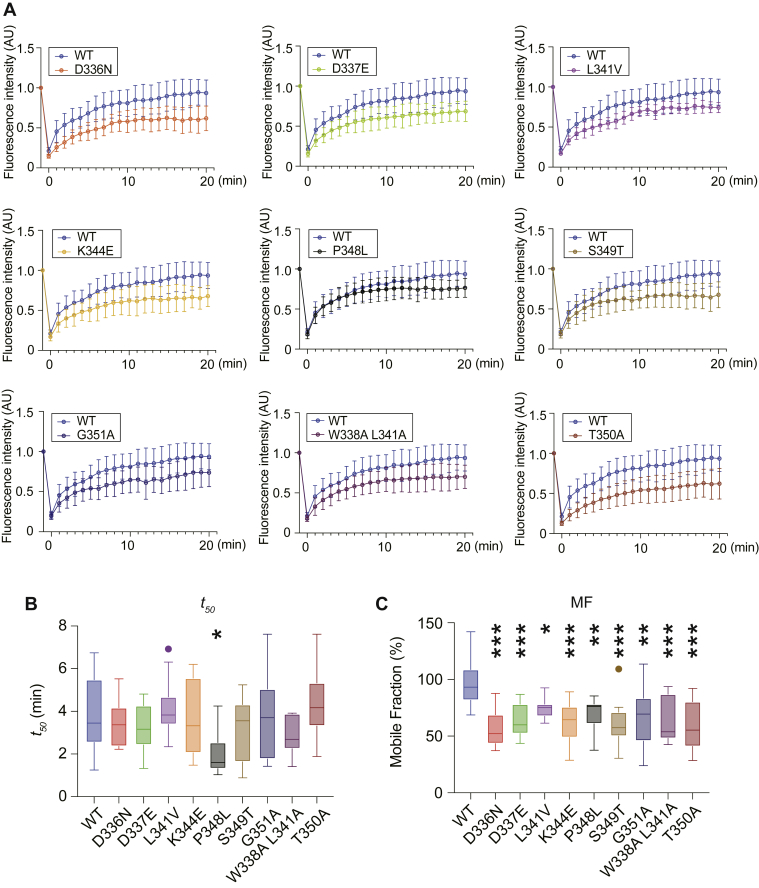
**Inner fluidity of disease-related p62 droplets.***A*, FRAP. Huh-1 cells were transfected by GFP-p62 and each mutant p62. At 24 h after transfection, the central portion of each p62 droplet labeled by GFP-wild-type or mutant p62 was photobleached, and the time of fluorescent recovery was measured. Data are means ± s.d of nonphotobleached (n = 10) and photobleached (n = 10) dots. Bar: 2 μm. *B* and *C*, *t*_*50*_ (*B*) and mobile fraction (*C*) of wild-type and mutant p62 liquid droplets. Data are means ± s.d. ∗*p* < 0.05 as determined by Dunnett’s test after ANOVA. *t*_*50*_ of droplets in wild-type p62-expressing cells and mutant-expressing cells was compared. Significant differences are shown for values of *t*_*50*_ and the mobile fraction of droplets in mutant p62-expressing cells and wild-type expressing cells.

Finally, to investigate the inner fluidity of p62 droplets, we measured fluorescence recovery after photobleaching (FRAP) of wild-type and each mutant p62 droplet (Figs. 7*A* and S3 and Movie S11–S20). Droplets with a diameter of 1.5 to 2 μm were measured. Molecular dynamics in FRAP experiments are commonly characterized by the half-time of recovery (*t50*) and the mobile fraction (34), and we calculated both values. Consistent with previous reports by us and other groups (17, 18, 21), the *t50* of GFP-p62 droplets was not very fast, at 3.74 ± 1.70 min (Figs. 7, *A* and *B*, and S3 and Movie S11). The *t50* of P348L, but not of other mutants, was slower than that of wild-type p62 (Fig. 7, *A* and *B*), suggesting slower dynamics of P348L. Remarkably, all p62 mutant droplets had a lower mobile fraction value than wild-type p62 droplets (Fig. 7*C*), indicating a lower number of moving p62 mutant molecules. This may be attributed to diverse factors such as the influx rate of cytoplasmic p62, the binding to client proteins such as KEAP1 and LC3, and the association of p62 with membranous structures. Evaluation of *t50* and the mobile fraction of whole p62 droplets indicated that droplets consisting of L341V, G351A, W338L L341A, and T350A showed no defects in p62 influx from the surrounding environment (Fig. S4). We also verified by immunofluorescent analysis that localization of KEAP1 and LC3 to the droplets was not correlated with defective inner fluidity (Fig. S5, *A* and *B*, Tables 2 and 3). Rather, the localization of KEAP1 and LC3 was associated with the *in vivo* interaction of KEAP1 with p62 (NanoBRET) and with autophagy-dependent degradation of p62, respectively (Table 2).

## Discussion

In the past few years, there has been increasing evidence for the formation and subsequent gel or aggregate transition of liquid droplets consisting of distinct ALS/FTD-related mutant proteins, including fused in sarcoma (FUS) (35, 36, 37), TAR-DNA-binding protein (TDP-43) (38), heterogeneous ribonucleoprotein A1 (hnRNPA1) (39), and Tau (40), as well as dipeptide repeat proteins derived from the *C9orf72* gene (41, 42). On the other hand, it has been unclear whether disease-related p62 mutants exhibit similar abnormalities. In this study, all ALS/FTD-associated p62 mutant proteins, which harbor missense mutations in the LIR and KIR, exhibited increased or decreased binding affinity to their binding partners, LC3 and KEAP1 (Figs. 2 and 3), and some resulted in suppression of p62-mediated NRF2 activation (Fig. 4) and p62 autophagic turnover (Fig. 5). Meanwhile, with the ability to form droplets (Fig. 6 and Movies S1–S10), all mutant p62 proteins consistently demonstrated reduced inner fluidity of the droplets (Fig. 7). This is probably due to improper assembly of multivalent macromolecules, including multidomain proteins and RNA, that result in sharp liquid–liquid demixing phase separations and the generation of micrometer-sized liquid droplets in aqueous solution (30). In the case of p62, the interaction with ubiquitinated proteins drives the liquid–liquid phase separation (17, 18), while a client protein, NBR1, affects the fluidity of p62 droplets (18, 24), the sequestration of KEAP1 into the droplets activates NRF2 (21, 25), and ATG proteins and LC3 promote autophagosome formation around the droplets (21, 43). The interaction of p62 with small noncoding RNA regulates the condensation of p62 (44). The imbalance of the association with such proteins and RNA due to p62 mutations may result in reduced inner fluidity.

In general, droplets with smaller size have higher circularity. As shown in Figure 6*B*, since droplets consisting of p62 mutants were smaller than those containing wild-type p62, the circularity of some mutant droplets such as L341V, P348L, and G351A might be higher despite their decreased fluidity. In addition, as mentioned above, p62 droplets in cells contain a variety of proteins, including ubiquitinated proteins, ATG proteins, and selective autophagy receptors (17, 18, 21, 24, 32), and also associate with and surround membranous structures (21, 24). These unique properties of wild-type p62 droplets might affect their circularity, raising the possibility that the droplets consisting of disease-related p62 mutants maintain higher circularity due to their lack of interaction with related proteins and of their association with membranes.

ALS and FTD are genetically linked to protein quality control factors in the proteasome and to autophagy pathways, such as UBQLN2, p62/SQSTM1, optineurin, and VCP, as well as to RNA-binding proteins (RBPs) such as FUS, TDP-43, Ewing sarcoma protein (EWS), TAT-binding protein-associated factor 15 (TAF15), hnRNPA1, and hnRNPA2/B1 (30). ALS/FTD-linked mutations in RBPs such as FUS and hnRNPA1, which phase-separate into stress granules, have been shown to accelerate the phase transition of these proteins from a dynamic liquid state to a solid fibril state (39, 45). In the case of FUS and hnRNPA1, it is plausible that the decreased fluidity of droplets consisting of p62 with disease-related mutations reflects their more solid state, leading to aggregate and/or fibril states. Though we observed short-term functional defects of only some p62 mutant proteins in terms of NRF2 activation and autophagic turnover, prolonged qualitative changes in p62 droplets should be accompanied by the same functional defects and/or aggregation-dependent gain-of-function cytotoxic effects. Indeed, aggregated structures such as the aggregate-prone Ape1 complex are not recognized as selective substrates due to failure of adaptor proteins to function with the aggregates (46). Under normal conditions, KEAP1 shuttles between the cytoplasm and p62 droplets (21), but it is sequestered in disease-associated p62 aggregates (21, 47, 48). Our hypothesis is also supported by a fact that missense mutations of *SQSTM1/p62* related to ALS/FTD have been identified throughout the gene, including in regions encoding the intrinsically disordered region and UBA domain, both of which are required for liquid droplet formation. p62 phase separation is dependent on p62 polymerization, the interaction between p62 and ubiquitin through the UBA domain, and the valence of the polyubiquitin chain (17). Weak and transient interactions between molecules with multivalent domains or intrinsically disordered regions are a driving force for liquid phase separation (30). In conclusion, we propose that the primary defect in ALS/FTD with p62 missense mutations does not involve selective autophagy or the antioxidative stress response, but rather a qualitative change in p62 liquid droplets, specifically reduced inner fluidity of the droplets followed by their aggregation.

## Experimental procedures

### Cell culture

Huh-1 (JCRB0199) and HEK293T (ATCC CRL-3216) cells were grown in Dulbecco’s modified Eagle medium (DMEM) containing 10% fetal bovine serum (FBS), 5 U/ml penicillin, and 50 μg/ml streptomycin. For overexpression experiments, Huh-1 and HEK293T cells were transfected with Lipofectamine 3000 (Thermo Fisher Scientific) and polyethyleneimine 25 kD linear (PEI, #23966, Polysciences), respectively. To generate *p62*-knockout HEK293T cells, *p62* guide RNA (CACCGTGAAGGCCTACCTTC) designed using the CRISPR Design tool (http://crispr.mit.edu/) was subcloned into pX330-U6-Chimeric_BB-CBh-hSpCas9 (#42230, Addgene), a human codon-optimized SpCas9 and chimeric guide RNA expression plasmid. The HEK293T cells were transfected with vector pX330 and cultured for 2 days. Thereafter, the cells were sorted and expanded. Loss of *p62* was confirmed by immunoblot analysis with anti-p62 antibody. All cells were authenticated by STR profile and tested for mycoplasma contamination.

### Isolation of mouse primary hepatocytes

Eight-week-old mice were anesthetized with isoflurane, and the mouse livers were perfused by reverse flow through the vena cava using 20 ml of ex-perfusate solution (pH 7.4, 140 mM NaCl, 5 mM KCl, 0.5 mM NaH_2_PO_4_, 10 mM HEPES, 4 mM NaHCO_3_, 50 mM glucose, and 0.5 mM GEDTA) and 30 ml of perfusate solution (pH 7.6, 70 mM NaCl, 6.7 mM KCl, 100 mM HEPES, 5 mM CaCl_2_, 35 units collagenase). After dissection, hepatocyte suspensions were washed three times in HBSS (Gibco, Thermo Fisher Scientific), and the cells were grown in collagen-coated plates with William’s Medium E (Gibco, Thermo Fisher Scientific) supplemented with 10% FBS.

### Adenovirus infection

Wild-type p62 and mutant adenoviruses were prepared using an Adenovirus Expression Vector Kit (TAKARA BIO). To express exogenous p62 and mutant p62 proteins in mouse primary culture hepatocytes, the cells were plated onto six-well dishes. At 24 h after plating, the medium was replaced with fresh medium containing adenovirus at a multiplicity of infection of 100 and cultured for 48 h.

### Generation of tet-ON cells

Tetracycline-mediated FLAG-tagged p62-expressing cell lines were generated using the reverse tet-regulated retroviral vector. A cassette consisting of the gene encoding the packaging signal (Ψ), the reverse tetracycline controlled transactivator (rtTA), the internal ribosome entry site from the ribosome (IRES), the puromycin resistance gene, a heptamerized tet operator sequence (tetO), the minimal human cytomegalovirus immediate-early promoter designated P_hcmv∗-1_ (CMV), and human p62 cDNA or various p62 mutants were cloned into a Moloney murine leukemia virus (M-MuLV) retroviral vector pLXSN backbone. Retrovirus packaging cells, PLAT-E, transfected with the vectors were cultured at 37 °C for 24 h. The medium was changed, then the virus producing PLAT-E was further incubated at 37 °C for 24 h. The viral supernatant was collected and used immediately for infection. p62-deficient Huh-1 cells were plated on 35-mm dishes in 3 ml growth medium at 24 h before infection. Just before infection, the medium was replaced with undiluted viral supernatant with 8 μg/ml polybrene (Sigma). Twenty-four hours later, the cells were introduced into the selection medium containing 2 μg/ml of puromycin dihydrochloride (Fujifilm Wako Pure Chemical Corporation). The cells remaining after 5 days were used in the experiments. To induce the expression of FLAG-p62, the cells were treated with 250 ng/ml of doxycycline (Sigma) for 24 h.

### Immunoblot analysis

Cells were lysed in ice-cold TNE buffer (50 mM Tris-HCl, pH 7.5, 150 mM NaCl, 1 mM EDTA) containing 1% Triton X-100 and protease inhibitors. Nuclear and cytoplasmic fractions were prepared using the NE-PER Nuclear and Cytoplasmic Extraction Reagents (Thermo Fisher Scientific). Samples were separated by SDS-PAGE and then transferred to polyvinylidene difluoride membranes. For immunoprecipitation analysis, cells were lysed by 200 μl of TNE, and the lysate was then centrifuged at 10,000*g* for 10 min at 4 °C to remove debris. Then, 800 μl of TNE and 1 μg of anti-FLAG antibody (M185-3L, Medical & Biological Laboratories) were added to the lysate, and the solution was mixed under constant rotation for 12 h at 4 °C. The immunoprecipitates were washed five times with ice-cold TNE. The complex was boiled for 10 min in SDS sample buffer in the presence of 2-mercaptoethanol to elute proteins and centrifuged at 10,000*g* for 5 min. Anti-S349-phosphorylated p62 polyclonal antibody was produced in rabbits using the peptide Cys+KEVDP(pS)TGELQSL as an antigen (20). Antibodies against p62 (PM066, Medical & Biological Laboratories), LC3B (#2775, Cell Signaling Technology), GAPDH (6C5, Santa Cruz Biotechnology), FIP200 (17250-1-AP, Proteintech Group), Lamin B (M-20, Santa Cruz Biotechnology), NRF2 (H-300; Santa Cruz Biotechnology), and KEAP1 (10503-2-AP, Proteintech Group) were purchased from the indicated suppliers and employed at 1:500 dilution. Blots were then incubated with horseradish-peroxidase-conjugated secondary antibody (Goat Anti-Mouse IgG (H + L), 115-035-166; Goat Anti-Rabbit IgG (H + L), 111-035-144; Goat Anti-Guinea Pig IgG (H + L); all from Jackson ImmunoResearch) and visualized by chemiluminescence.

### NanoBRET assay

p62 was subcloned into the EcoRI and XbaI sites of pHTN HaloTag CMV-neo vector (G772A, Promega). KEAP1 was subcloned into the NheI and XbaI sites of pNLF1-C (CMV/Hygro) vector (N136A, Promega), and LC3B and FIP200 into the EcoRI and XbaI and EcoRI and NotI sites of pNLF1-N (CMV/Hygro) vector (N1351, Promega), respectively. HEK293T *p62*-knockout cells were cotransfected with Halo-tagged p62 and NanoLuc-tagged KEAP1, LC3B, or FIP200 by polyethylenimine 25 kD linear (PEI, #23966, Polysciences). Twenty-four hours after the transfection, the cells were seeded onto a white 96-well plate (152028, Thermo Fisher Scientific) at 2 × 10^4^ cells/well and labeled with Halo tag NanoBRET 618 ligand (G9801, Promega) for 24 h. Thereafter, the cells were incubated with 50 μl of DMEM containing 4% FBS and NanoBRET substrate (N1571, Promega) for 5 min. The BRET signal was measured by a GloMax Discover Microplate Reader (GM3000, Promega) using a 460-nm filter for the donor signal and a 610-nm filter for the acceptor signal. The BRET ratio was determined by dividing the acceptor signal by the donor signal.

### Immunofluorescence microscopy

Cells grown on coverslips were fixed in 4% paraformaldehyde in PBS for 10 min, permeabilized with 0.1% Triton X-100 in PBS for 5 min, blocked with 0.1% (w/v) gelatin (Sigma-Aldrich) in PBS for 45 min, and then incubated overnight with primary antibodies diluted 1:200 in gelatin/PBS. After washing, cells were incubated with Goat Anti-Guinea Pig IgG (H + L) Cross-Adsorbed Secondary Antibody, Alexa Fluor 488 (A11073, Thermo Fisher Scientific) and Goat Anti-Mouse IgG (H + L) Highly Cross-Adsorbed Secondary Antibody, Alexa Fluor 647 (A21236, Thermo Fisher Scientific) at a dilution ratio of 1:1000 for 60 min. Cells were imaged using a confocal laser-scanning microscope (Olympus, FV1000) with a UPlanSApo × 60 NA 1.40 oil objective lens.

### Determination of the circularity, size, and number of p62 droplets

FLAG-p62 proteins were expressed in *p62*-knockout Huh-1 cells using an adenovirus system. The cells were fixed with 4% paraformaldehyde in PBS for 10 min, permeabilized with 0.1% Triton X-100 in PBS for 5 min, blocked with 0.1% (w/v) gelatin (Sigma-Aldrich) in PBS for 45 min, and then incubated overnight with p62 antibody diluted 1:200 in gelatin/PBS. After washing, cells were incubated with Goat Anti-Guinea Pig IgG (H + L) Cross-Adsorbed Secondary Antibody at a dilution ratio of 1:1000 for 60 min. After nuclei were stained with Hoechst, they were imaged using a Confocal Quantitative Image Cytometer (CQ1, Yokogawa). To quantify the circularity, size, and number of p62 droplets, the images of over 300 cells with p62 signals were analyzed by Cellpathfinder software (Ver.R3.04, Yokogawa). To analyze p62 droplets exclusively, the p62-positive structures with a diameter of 1.02 to 5.78 μm were identified.

### Fluorescence recovery after photobleaching

To construct GFP-tagged p62 constructs, p62 was subcloned into the BglII and KpnI sites of pEGFP-C1 plasmid (6084-1, Clontech). To measure the fluorescence recovery after photobleaching (FRAP), cells were grown in 35-mm glass-base dishes. GFP-p62 was transfected into the cells with Lipofectamine 3000 (Thermo Fisher Scientific) for 24 h. p62-positive droplets with a diameter of 1.6 to 1.9 μm were bleached using a laser intensity of 10% at 488 nm, and then the recovery was recorded for the indicated times. Olympus FV31S-SW software (version: 2.4.1.198) was used for image collection and analysis. After image acquisition, contrast and brightness were adjusted using Photoshop 2021v25.0 (Adobe). The mobile fraction was calculated from ten measurements by the following equation: Mf = (F_∞_−F_0_)/(F_i_−F_0_) where Mf is the mobile fraction, F_∞_ is the fluorescence intensity after full recovery (plateau), F_i_ is the initial fluorescence intensity prior to bleaching, and F_0_ is the fluorescence intensity immediately after bleaching. The half-time (*t*_*50*_) of fluorescence recovery was calculated from ten measurements by curve fitting using the one-phase decay model of GraphPad PRISM 9 (GraphPad Software).

### Quantitative real-time PCR (qRT-PCR)

cDNAs were synthesized with 1 μg of total RNA using FastGene Scriptase Basic cDNA Synthesis (NE-LS62, NIPPON Genetics). Quantitative PCR was performed with TaqMan Fast Advanced Master Mix (444556, Thermo Fisher Scientific) on a QuantStudio 6 Pro (A43180, Thermo Fisher Scientific). Signals were normalized against *Gusb* (β-glucuronidase). Predesigned TaqMan Gene Expression Assays, including a primer set and TaqMan probe (Gusb, Mm01197698_m1; Nqo1, Mm01253561_m1; Ugdh, Mm00447643_m1; and Gstm1, Mm00833915_g1) were purchased from Thermo Fisher Scientific.

## Data availability

The authors declare that the data supporting the findings of this study are available within the article and its supporting information.

## Supporting information

This article contains supporting information.

## Conflict of interests

We declare that we have no competing financial interests.
