# Supplementary material for: Phase-separated protein droplets of amyotrophic lateral sclerosis-associated p62/SQSTM1 mutants show reduced inner fluidity
Source: J Biol Chem. 2021 Nov 12;297(6):101405. doi: 10.1016/j.jbc.2021.101405 (PMC8649403; doi:10.1016/j.jbc.2021.101405)
Supplement: Figures S1–S5 [file mmc1.pdf]

Supporting information

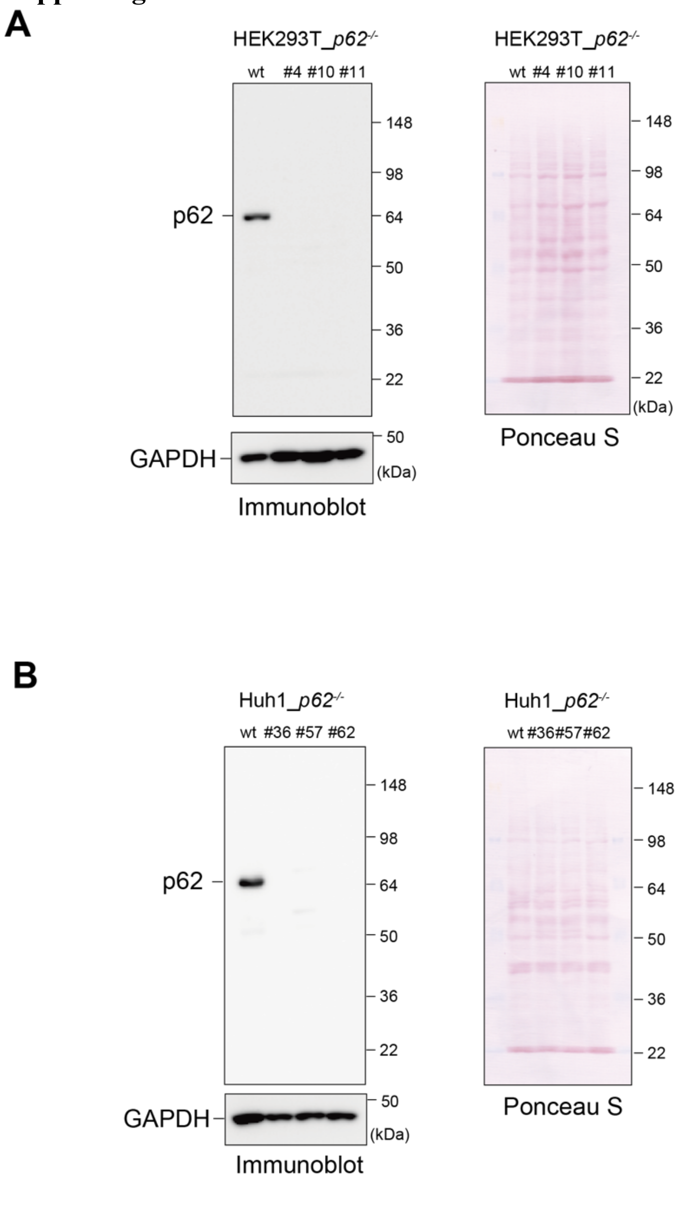

Supplementary Figure S1

Supplementary Figure S1 Generation of *p62*-deficient cells
(**A** and **B**) Immunoblot analysis. Parental and *p62*-deficient HEK293T (A) and Huh 1(B) cells were lysed, and then cell lysates were subjected to SDS-PAGE followed by immunoblotting with the indicated antibodies. Right panels show Ponceau-S staining.

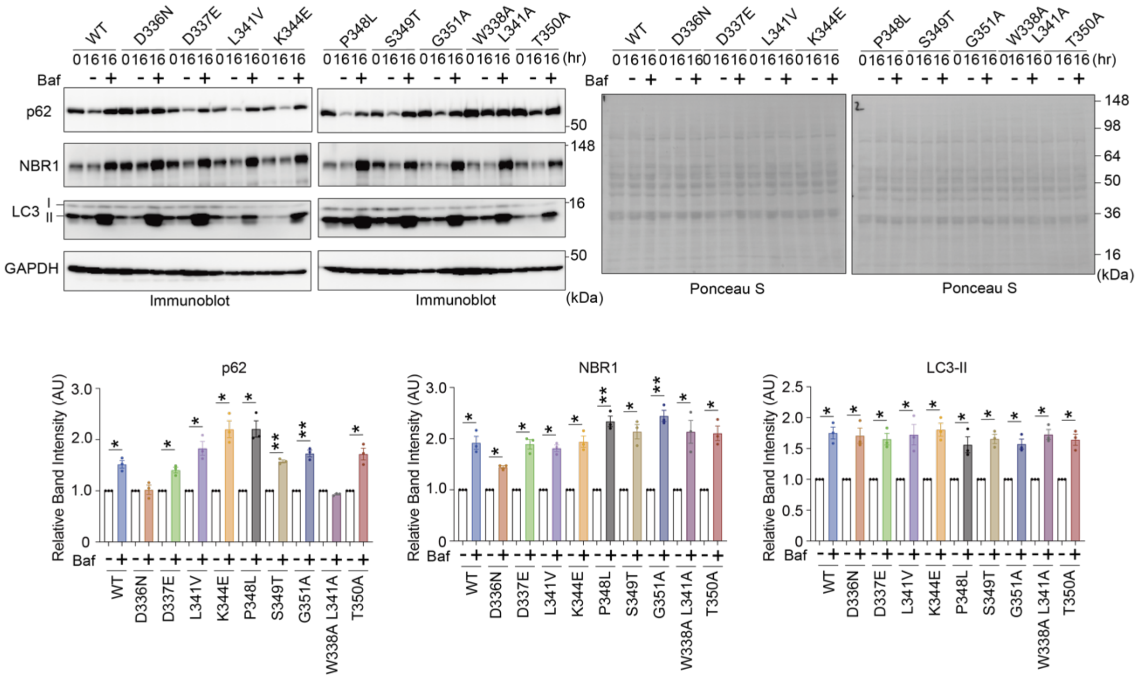

Supplementary Figure S2

Supplementary Figure S2 Flux assays of p62, NBR1, and LC3-II

In *p62*-knockout Huh-1 cells, FLAG-p62 or each disease-related mutant was expressed by the treatment of doxycycline for 24 h, then the doxycycline-containing medium was removed and cells were cultured with media in absence or presence of bafilomycin A_1 (Baf) for 16 h. The cell lysates were prepared and subjected to SDS-PAGE followed by immunoblotting with the indicated antibodies. Data are representative of three separate experiments. Bar graphs indicate the quantitative densitometric analysis of the indicated proteins relative to whole proteins estimated by Ponceau-S staining (n = 3). Data are means ± s.d. $*p < 0.05$ and $**p < 0.01$ as determined by two-sided Welch's *t*-test. The levels of each p62 protein at 16 h after removal of DOX were compared with those of p62 proteins treated with Baf.

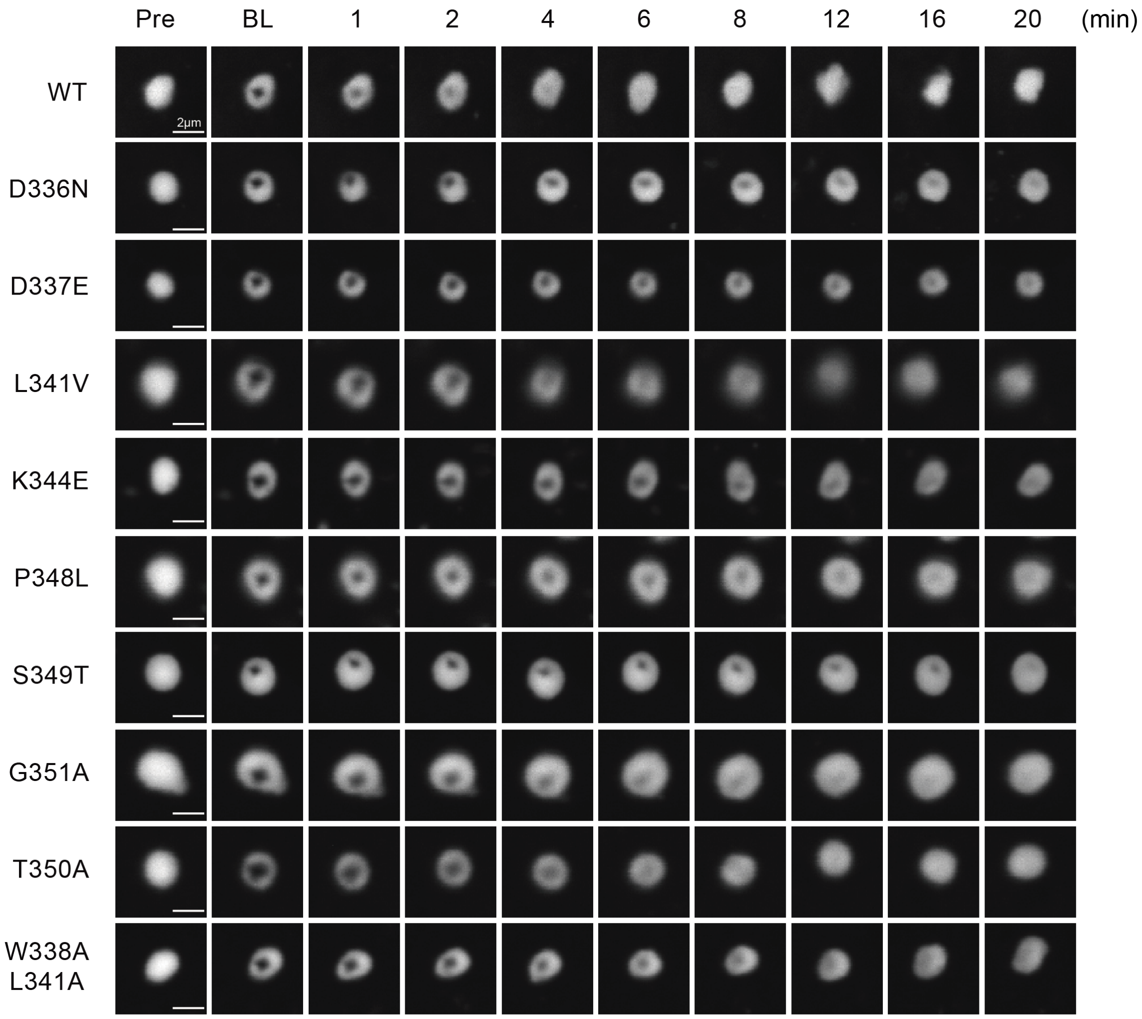

Supplementary Figure S3

Supplementary Figure S3 FRAP of disease-related p62 droplets
Time-lapse microscopic analysis. Wild-type p62 and each mutant GFP-p62 was transfected into *p62*-deficient Huh-1 cells. Twenty-four hours after transfection, the GFP-positive p62 droplets were photobleached, and the time of fluorescent recovery was measured. Pre; Pre-bleaching, BL; Bleaching. Bar: 2 μm.

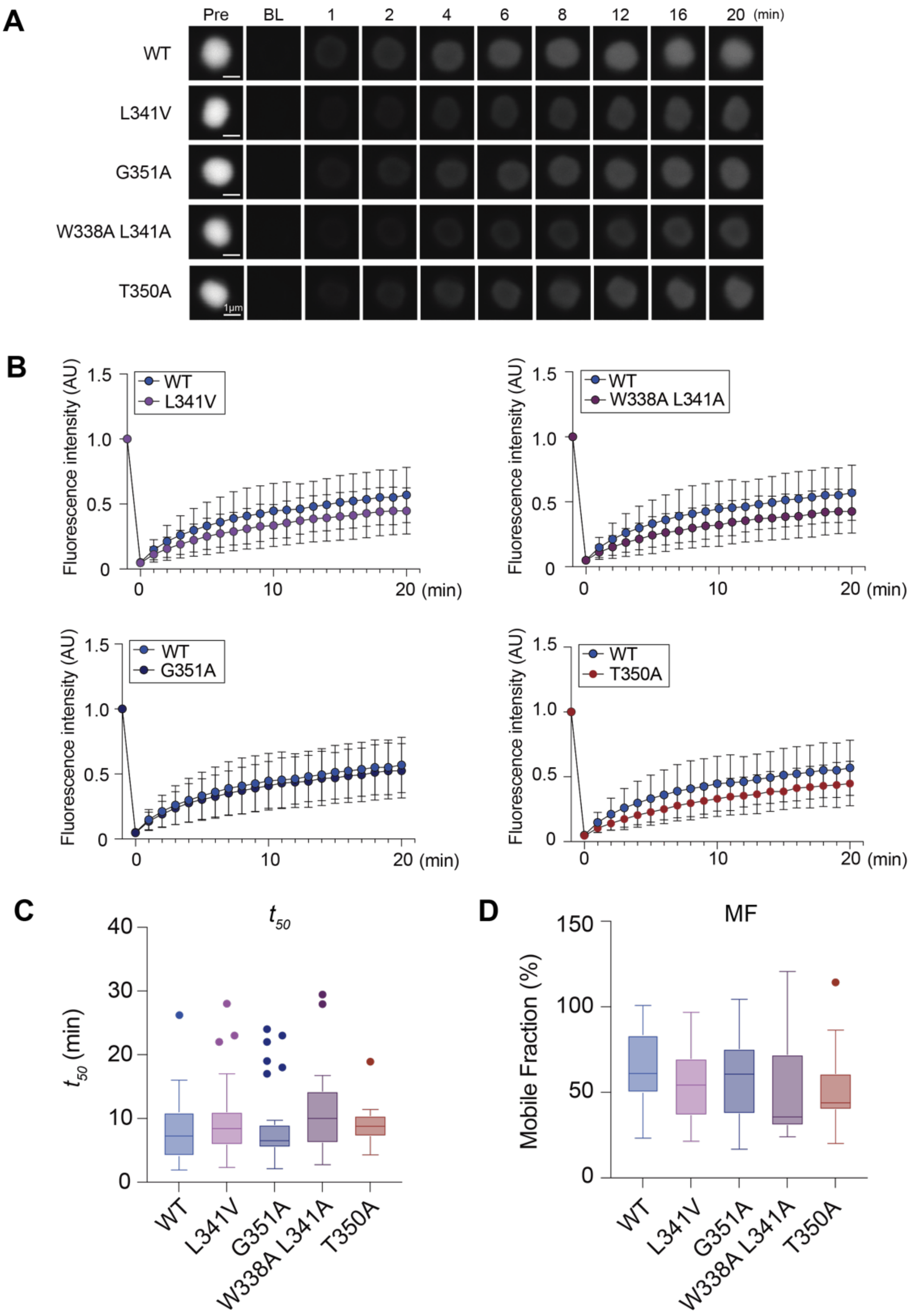

Supplementary Figure S4

Supplementary Figure S4 Influx of cytoplasmic disease-related p62 mutants into droplets
(**A-B**) FRAP. Wild-type p62 and each mutant GFP-p62 was transfected into *p62*-deficient Huh-1 cells. Twenty-four hours after transfection, whole GFP-positive p62 droplets were photobleached, and the time of fluorescent recovery was measured. Pre; Pre-bleaching, BL; Bleaching. Bar: 1 μm.
(**C-D**) t_{50} (C) and mobile fraction (D) of wild-type and mutant p62 liquid droplets. Statistical analysis was done by Dunnett's test after ANOVA. Significant differences are shown between values for wild-type p62-expressing cells and mutant-expressing cells.

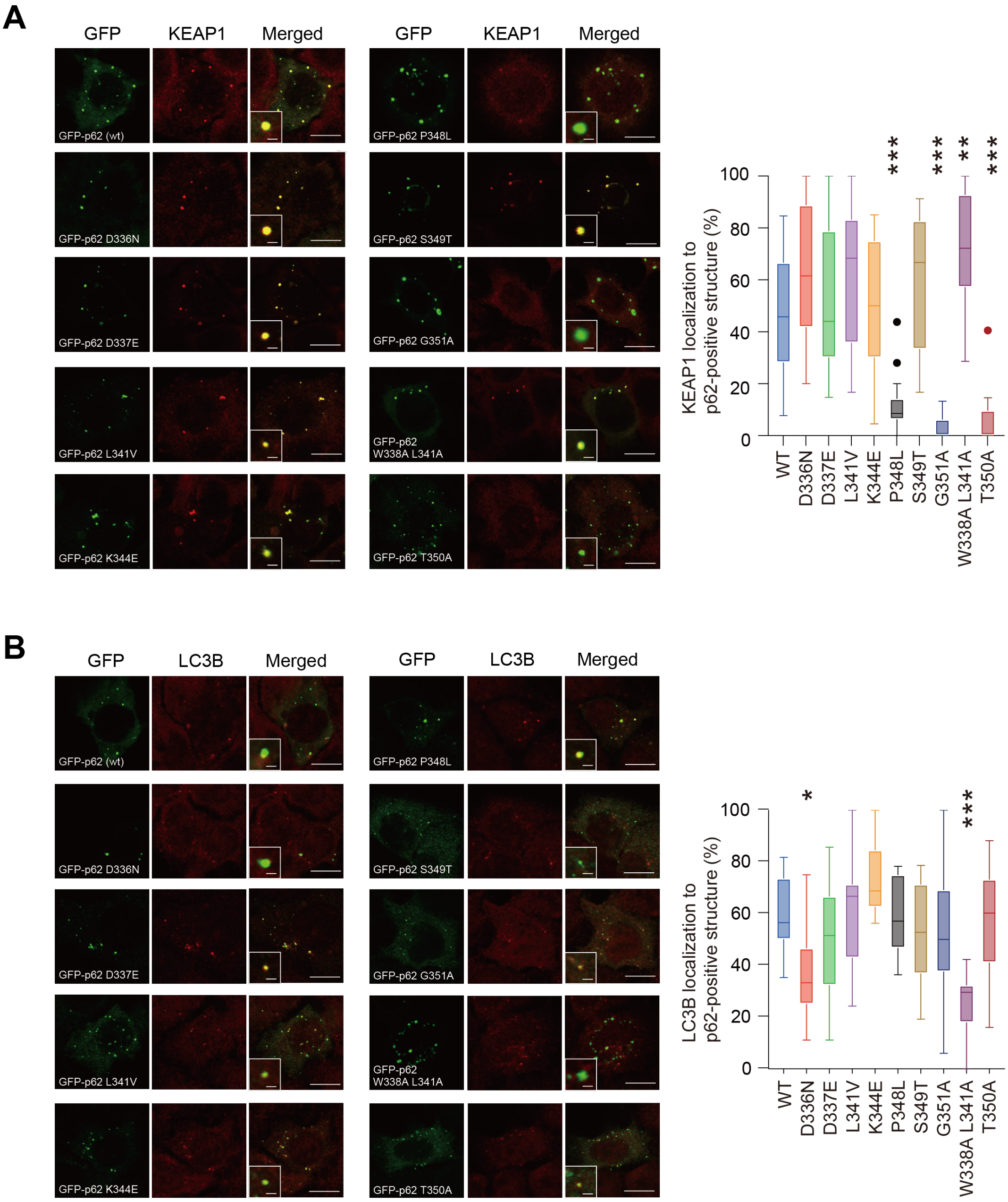

Supplementary Figure S5

Supplementary Figure S5 Localization of LC3 and KEAP1 with disease-related p62 droplets

(**A** and **B**) Immunofluorescence microscopy. *p62*-deficient Huh-1 cells were transfected with GFP-tagged wild-type p62 or disease-related mutants. Twenty-four hours after transfection, the cells were immunostained with LC3B (A) or KEAP1 (B) antibody. Scale bars: overviews, 10 µm; insets, 1 µm. Data are means ± s.d. **p* < 0.05, ***p* < 0.01*** and *p* < 0.001 as determined by Dunnett's test after ANOVA. Significant differences are shown for the values of LC3B or KEAP1 colocalized with mutant p62 droplets versus those of wild-type p62 droplets.

Supplementary Movies S1-S10 Time-lapse video of disease-related p62 liquid droplets
Time-lapse video microscopic analysis of p62-positive structures labelled with GFP-p62 (S1), D336N (S2), D337E (S3), L341V (S4), K344E (S5), P348L (S6), S349T (S7), G351A (S8), W338A L341A (S9), and T350A (S10).

Supplementary Movies S11-S20 Time-lapse video of disease-related p62 liquid droplets after photobleaching
Time-lapse video microscopic analysis of p62-positive structures labelled with GFP-p62 (S11), D336N (S12), D337E (S13), L341V (S14), K344E (S15), P348L (S16), S349T (S17), G351A (S18), W338A L341A (S19), and T350A (S20) after photobleaching.
